# Poultry consumption and prostate cancer risk: a meta-analysis

**DOI:** 10.7717/peerj.1646

**Published:** 2016-02-02

**Authors:** Qian He, Zheng-ce Wan, Xiao-bing Xu, Jing Wu, Guang-lian Xiong

**Affiliations:** Key Laboratory of Environment and Health, Ministry of Education & Department of Epidemiology and Biostatistics, School of Public Health, Tongji Medical College, Huazhong University of Science and Technology, Wuhan, China

**Keywords:** Poultry, Prostate cancer, Risk, Meta-analysis

## Abstract

**Background.** Several kinds of foods are hypothesized to be potential factors contributing to the variation of prostate cancer (PCa) incidence. But the effect of poultry on PCa is still inconsistent and no quantitative assessment has been published up to date. So we conducted this meta-analysis to clarify the association between them.

**Materials and Methods**. We conducted a literature search of PubMed and Embase for studies examining the association between poultry consumption and PCa up to June, 2015. Pooled risk ratio (RR) and corresponding 95% confidence interval (CI) of the highest versus lowest poultry consumption categories were calculated by fixed-effect model or random-effect model.

**Results.** A total of 27 (12 cohort and 15 case-control) studies comprising 23,703 cases and 469,986 noncases were eligible for inclusion. The summary RR of total PCa incidence was 1.03 (95% CI [0.95–1.11]) for the highest versus lowest categories of poultry intake. The heterogeneity between studies was not statistically significant (*P* = 0.768, *I*^2^ = 28.5%). Synthesized analysis of 11 studies on high stage PCa and 8 studies on chicken exposure also demonstrated null association. We also did not obtain significant association in the subgroup of cohort study (RR = 1.04, 95% CI [0.98–1.10]), as well as in the subgroups of population-based case-control study and hospital-based case-control study. Then the studies were divided into three geographic groups: Western countries, Asia and South America. The pooled RRs in these areas did not reveal statistically significant association between poultry and PCa.

**Conclusions.** This meta-analysis suggests no association between poultry consumption and PCa risk. Further well-designed studies are warranted to confirm the result.

## Introduction

Prostate cancer (PCa) is the second most common form of cancer and the fifth leading cause of cancer death among males. The age-standardized incidence rate varies up to 25-fold worldwide, ranging from 111.6 in Oceania to 4.5 in South-Central Asia per 100,000 (Ferlay et al., 2015). Dietary habits and lifestyles, besides genetic susceptibility and screening intensity, were thought to be etiological factors accounting for the substantial geographic variation. Migration researches have demonstrated the influence of diet and lifestyle on PCa years ago. The incidence of immigrants usually tended to deviate from the rate in the country of origin and shift toward the rate in the host country (Mousavi, Sundquist & Hemminki, 2013; Shimizu et al., 1991). Also adopting Western diet was widely perceived as a contributor to higher risk of PCa, whereas Mediterranean diet and Asian diet were likely to have protective effect (Lin, Aronson & Freedland, 2015). Hence, accumulated studies linking foods and nutrients with PCa risk have been stimulated (Lin, Aronson & Freedland, 2015; Masko, Allott & Freedland, 2013).

Meat attracted nutritionists’ concern for hazardous component. Researchers have found that meat cooked in high temperature contained considerable amount of mutagens. The hazardous component evoked the hypothesis that meat consumption would be in positive association with elevated PCa risk. Red meat and processed meat were proved to support the hypothesis and lower consumption was thus recommended (Mandair et al., 2014). The role of poultry, counterpart of red meat, has also been evaluated in a number of studies, but the evidence was inconclusive. For one thing, some studies was in favor of the hypothesis. Stott-Miller and colleagues’ (2013) study on fried chicken demonstrated that fried chicken would place individuals at heightened risk of PCa. Also statistics of the United States revealed strong positive correlation between PCa mortality and per capita consumption of chicken (Colli & Colli, 2005). There was also study claiming decreased risk when high amount of poultry intake compared with low intake (Hu et al., 2008). However, a large number of studies observed null association between them. A substitution model and addition model in a cohort study also failed to obtain statistically significant relationship (Daniel et al., 2011a).

To our knowledge, no article that attempted to quantitatively synthesize the effect of poultry consumption on PCa risk has been published up to now. Although there were two existing reports involving this topic (Norat et al., 2014; Cui et al., 2014), both of them were inaccessible in global literature databases and did not include all available studies. One report containing 12 studies was an internal review from the Continuous Update Project Panel, and the other was a qualitative review retrieved from China National Knowledge Infrastructure database. Herein, we conducted this meta-analysis to comprehensively examine the consistency and strength of the association. We sought to evaluate the relation of histology-specific PCa incidence and type-specific poultry exposure. Stratified analyses based on study design and geographic region were also performed.

## Materials and Methods

### Literature search

To ensure the rigor of this meta-analysis, we designed and reported it adhering to the criteria set out by PRISMA statement (Moher et al., 2009). To identify eligible studies, a comprehensive literature search was conducted in PubMed and Embase databases without language restriction up to June, 2015. The following subject heading terms, along with synonymic free text words, were used: “poultry,” “chicken,” “turkey,” “duck,” “geese,” “white meat,” “dietary proteins,” and “prostate cancer.” The detailed search strategies for the two databases were presented in Table S1. References of articles and reviews of interest were also scanned for additional relevant studies.

### Inclusion and exclusion criteria

Studies were eligible for inclusion if they satisfied the following criteria: (1) used a cohort or case-control design; (2) contained data on PCa incidence; (3) identified poultry as exposure, including chicken, turkey, processed poultry etc.; and (4) provided the extreme categories of poultry consumption and corresponding relative risks with 95% confidence intervals (CI).

Articles in inappropriate formats, such as review, conference paper, editorial and letter, were not considered. Cross-sectional study, ecologic study and non-human research were excluded for unavailable effect size estimate. Studies that reported data for broad classification of meat, such as white meat, were excluded. If there was overlapping period of data collection in the same setting in several articles, only the most recent and complete one was chosen.

### Data extraction and quality assessment

Data were extracted by two independent researchers (Wan ZC and Xu XB), and the discrepancies between them were resolved by a third contributor. For each study meeting the inclusion criteria, the following characteristics were collected: author, year, study location, study design, sample size (i.e., number of incident cases and controls/participants), study period, duration of follow-up for cohort studies, participants’ age at baseline, exposure, analytical comparison (i.e., the exposure contrast), the relative risk and 95% CI that reflected the maximum extent of adjustment for potential confounders, and variables adjusted for or matched by in the statistical analysis. If an article separately reported effect size estimates on total PCa and high stage PCa, both of them were extracted. Crude effect size was calculated by available data if it was absent in the original paper. When the full article or necessary data were unavailable, we attempted to contact the author by email to ask for sufficient information.

Quality assessment of included studies was evaluated using the Newcastle-Ottawa Quality Assessment Scale (Wells et al., 2000). The scale was a validated semi-quantitative assessment technique for non-randomized studies in meta-analysis. A nine-point system was used to allocate points to studies based on three criteria: four for participant selection, two for comparability of study groups, and three for assessment of outcome or exposure.

### Statistical analysis

Statistical analysis was based on comparison of the highest poultry intake category with the lowest one. As different studies reported different cutoffs (e.g., quintiles, quartiles, tertiles, yes or no etc.), the categories were extracted from the corresponding exposure groups in original articles without unification. The measure of interest was risk ratio (RR) with 95% CI. Hazard ratio was directly considered as RR. Given that PCa was a rare outcome and thus odds ratio in case-control study mathematically approximated to RR (Greenland, 1987), odds ratio was also reported as RR for simplicity. Several articles provided two or more statistical models, and the RRs in the greatest degree of adjustment were used. The effect size estimates were absent in two original papers (Joshi et al., 2012; Lee et al., 1998), and crude values were calculated.

The heterogeneity among studies was estimated by Cochran *χ*^2^-based *Q* test and *I*-square (*I*^2^) statistic. Heterogeneity was considered significant if *P* value was below 0.05 for *Q* statistic or *I*^2^ value was above 75%. *I*^2^ statistic represented the proportion of variation across studies due to between-study heterogeneity, and the values of 25%, 50% and 75% were regarded as cut-off points for low, moderate and high degrees of heterogeneity (Higgins et al., 2003). To calculate the summary estimate and 95% CI, we used a fixed-effect model (Mantel-Haenszel method) when the heterogeneity was negligible, or random-effect model (DerSimonian and Laird method) when the heterogeneity was significant (Lau, Ioannidis & Schmid, 1997). The individual and overall effects were illustrated by forest plot. Univariate meta-regression analysis (significant at *P* < 0.2) was conducted to explore source of heterogeneity if it was significant. The following pre-specified study-level covariates were assessed: type of exposure (total poultry and chicken only), study design (cohort study, retrospective case-control study with population controls and that with hospital controls), geographic region (Western countries, Asia and South America), and adjustment for family history of PCa (yes and no). Tau-square (*τ*^2^) statistic would be used as the measurement of proportion of heterogeneity that covariate accounted for. In order to assess the influence of individual study on the overall estimate, sensitivity analysis was carried out by omitting one study at a time and calculating the summary effect for the remainders. Publication bias was evaluated by funnel plot, and quantified by Egger’s test (Egger et al., 1997) and Begg’s test (Begg & Mazumdar, 1994) for funnel plot asymmetry.

**Figure 1:**
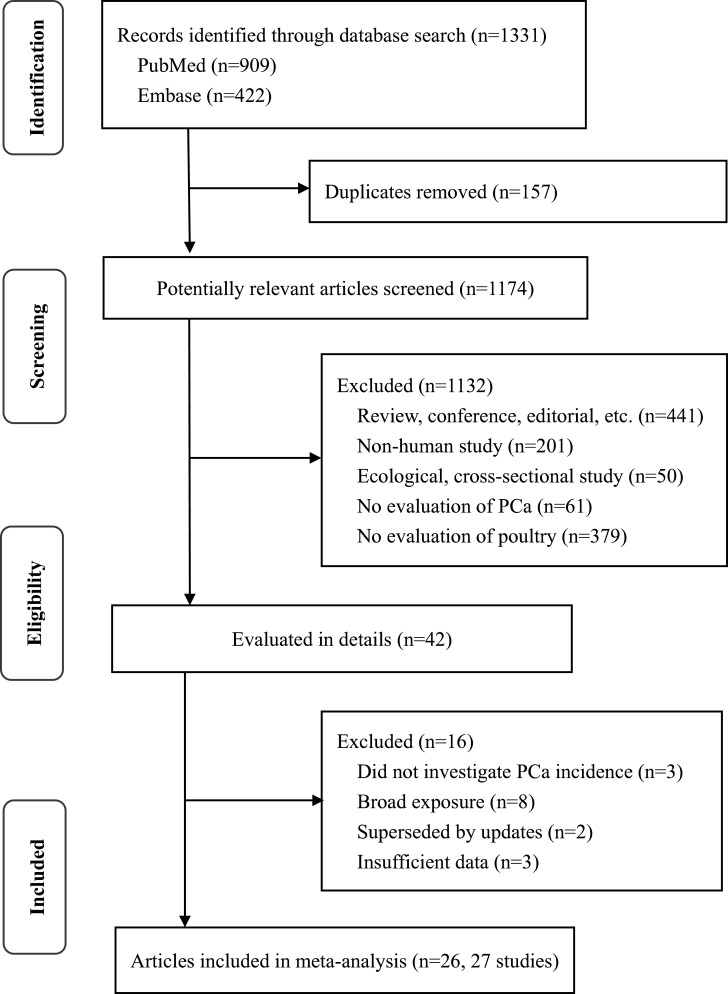
Flowchart of the literature search.

We also pooled the studies on high stage PCa and the studies on chicken exposure respectively. Stratified analyses by study design and geography were subsequently done. A two-tailed *P* value less than 0.05 was considered to be statistically significant for all tests except for meta-regression. All statistical analyses were performed using STATA12.0 (Stata Corporation, College Station, Texas, USA).

## Results

### Literature search

Detailed process of literature selection was described in Fig. 1. Briefly, our search strategy initially yielded 1,331 records, and 157 of them were duplicates. After exclusion of articles that were irrelevant to our object and in inappropriate formats and designs, 42 remainder articles seemed to fulfill the inclusion criteria of this meta-analysis. When evaluated the full texts, we excluded 16 articles as follows. Three articles did not focus on PCa incidence and eight articles merely reported data for broad exposure. Two articles (Giovannucci et al., 1993; Michaud et al., 2001) were superseded by a more recent and complete article in the same setting (Richman et al., 2011). One article (Ross et al., 1987) was perused but excluded for missing CI. Another cohort study (Schuurman et al., 1999) reporting RR for 25 g per day increase was excluded, because there was insufficient data to convert the effect to RR for extreme comparison groups. Besides, Daniel and colleagues’ (2011a) study presented additive effect but not effect of actual poultry consumption, and thus it was excluded. Whereas, an article (Rodriguez et al., 2006) separately providing data on American Whites and Blacks was considered as two independent studies. Thus, the meta-analysis ultimately included 27 independent studies in 26 articles (Allen et al., 2008; Allen et al., 2004; Amin et al., 2008; Bosetti et al., 2004; Deneo-Pellegrini et al., 1999; Deneo-Pellegrini et al., 2012; Hsing et al., 1990; Hu et al., 2008; Iso & Kubota, 2007; Jain et al., 1999; Joshi et al., 2012; Koutros et al., 2008; Le Marchand et al., 1994; Lee et al., 1998; Li et al., 2008; Mahmood et al., 2012; McCann et al., 2005; Mills et al., 1989; Park et al., 2007; Punnen et al., 2011; Richman et al., 2011; Rodriguez et al., 2006; Rohrmann et al., 2007; Rovito et al., 2005; Stott-Miller, Neuhouser & Stanford, 2013; Sung et al., 1999).

### Study characteristics and quality assessment

Table 1 showed the main characteristics of studies included in this meta-analysis. We finally identified 12 prospective cohort studies, 6 population-based case-control studies and 9 hospital-based case-control studies. A total of 14,202 cases and 470,997 participants from cohort studies, and 9,501 cases and 13,191 controls from case-control studies enrolled in the present meta-analysis. More than half of the studies were conducted in North America and Europe, including 14 in the United States, 3 in Canada, 1 in Italy, and 1 in eight European countries. There were six researches in Asia, including 2 in Japan, 2 in mainland China, 1 in Taiwan, and 1 in Pakistan. The other two studies were performed in Uruguay in South America. Most of the participants were in their middle or old age, roughly ranging from 45 to 75 years old at baseline. Structured food frequency questionnaire based interview was the approach to assess exposure in all studies. The exposure was either chicken or total poultry. Dietary intake in the articles was primarily assessed in scales of frequency or gram. In most studies, the poultry consumption was about 1–3 times/week or 250–300 g/week in the highest groups, and about 0–1 time/week or 50–100 g/week in the lowest groups. Relative risk estimate was adjusted for or matched by age and other confounders in all original reports except for the two recalculated studies. Assessment of study quality yielded a score of 5–8 on the scale of the nine-point scoring system for each study. The average scores of cohort study and case-control study were 7.9 and 6.9 respectively (Table S2).

**Table 1:** Characteristics of included studies of poultry consumption and PCa risk.

Author, year, location, design	Cases/ controls, *n*	Study period	Age at baseline (y)	Exposure comparison	Relative risk (95% CI)^a^	Statistical adjustment
Mills et al. (1989), USA, Cohort	180/35,000	1976–1982, 6y	Range: ≥25	Poultry; 3rd tertile vs. 1 (≥1 vs. 0 times/w)	1.34 (0.82–2.19)	Age
Hsing et al. (1990), USA, Cohort	149/17,633	1966–1986, 20y	Median: 51; Range: ≥35	Chicken ; 4th quartile vs. 1 (>4 vs. ≤0.5 times/m)	H: 0.90 (0.40–1.80)	Age, smoking
Le Marchand et al. (1994), USA, Cohort	198/8,881	1975/1980–1989, 6y	Range: ≥45	Poultry; 4th quartile vs. 1 (>139 vs. ≤45 g/w)	1.10 (0.70–1.70)	Age, ethnicity, income
Lee et al. (1998), China, PCC	133/265	1989–1992	Range: 50–89	Poultry; 3rd tertile vs. 1 (NA)	1.12 (0.41–3.52)	Crude estimate calculated by original data
Deneo-Pellegrini et al. (1999), Uruguay, HCC	175/233	1994–1997	Range: 40–89	Poultry; 4th quartile vs. 1 (≥53 vs. ≤12 servings/y)	1.30 (0.70–2.40)	Age, residence, urban/rural status, education, family history, BMI , total energy intake
Jain et al. (1999), Canada, PCC	617/636	1989–1993	Mean: case 69.8, control 69.9	Chicken; 4th quartile vs. 1 (>44.6 vs. <9.9 g/d)	1.02 (0.77–1.34)	Age, total energy, vasectomy, ever-smoked, marital status, study area, BMI, education, ever-used multivitamin supplements in previous 1y, grains, fruit, vegetables, total plants, total carotenoids, folic acid, dietary fiber, conjugated linoleic acid, vitamin E, vitamin C, retinol, total fat, linoleic acid
Sung et al. (1999), Taiwan, HCC	90/180	1995–1996	Range: ≥50	Chicken; yes vs. no (≥1 vs. 0 times/w)	1.73 (0.90–3.31)	Matched by age, treatment hospital, date of admission
Allen et al. (2004), Japan, Cohort	196/18,115	1963/1965/1979–1996, 16.9y	Mean 51; range: 18–99	Chicken; 3rd tertile vs. 1 (>4 vs. <2 times/w)	0.77 (0.19–3.10)	Age, calendar period, city of residence, radiation dose, education
Bosetti et al. (2004), Italy, HCC	1,294/1,451	1991–2002	Median: case 66, control 63; range: 46–74	Poultry; 5th quintile vs. 1 (median 3 vs. 0.5 servings/w)	1.26 (0.98–1.61)	Age, study center, education, social class, BMI, family history, total calorie intake
McCann et al. (2005), USA, PCC	433/538	1986–1991	NA	Poultry; 4th quartile vs. 1 (>34 vs. ≤13 g/d)	0.97 (0.67–1.39)	Age, education, BMI, smoking status
Rovito et al. (2005), USA, HCC	152/161	1998–2001	Mean (s.d.): case 63.07 ± 10.9, control 66.57 ± 9.0; range: 31–88	Poultry; 3rd tertile vs. 1 (>38 vs. <15 g/d)	0.62 (0.34–1.11)	Race, family history; matched by age
Rodriguez et al. (2006), USA, Cohort (Whites)	5,028/64,897	1992–2001, 9y	Range: 50–74	Poultry; 4th quartile vs. 1 (≥279 vs. <91 g/w)	1.00 (0.90–1.10) H: 0.70 (0.40–1.10)	Age, total calorie intake, BMI, level of education, family history, history of PSA testing, history of diabetes
(Blacks)	85/693				0.70 (0.40–1.30)	
Iso & Kubota (2007), Japan, Cohort	169/46,465	1990–2003, 12y	Range: ≥40	Chicken; 3rd tertile vs. 1 (≥3 vs. <1 times/w)	H: 1.33 (0.81–2.21)	Age
Park et al. (2007), USA, Cohort	4,404/82,483	1993–2002, 8y	Range: ≥45	Poultry; 5th quintile vs. 1 (median 39.9 vs. 5.9 g/1000 kcal/d)	1.01 (0.92–1.12) H: 1.06 (0.88–1.28)	Age, time on study, ethnicity, family history, education, BMI, smoking status, energy intake
Rohrmann et al. (2007), USA, Cohort	199/3,892	1989–2004, 15y	Mean: 53.8; range: ≥35	Poultry; 3rd tertile vs. 1 (≥5 vs. ≤1 times/w)	1.14 (0.77–1.70) H: 0.60 (0.24–1.49)	Age, energy intake, saturated fat intake, tomato products intake, BMI at age 21
Allen et al. (2008), 8 European countries, Cohort	2,727/142,251	1989–2007, 8.7y	Median: 52; 5th–95th percentile: 33–67	Poultry; 5th quintile vs. 1 (median 32 vs. 9 g/d)	1.12 (0.98–1.27)	Age, center, education, marital status, height, weight, energy intake
Amin et al. (2008), Canada, HCC	386/268	2003–2006	Mean (s.d.): 64.5 ± 8.3	Chicken; 4th quartile vs. 1 (4 vs. 1 servings/w)	1.26 (0.58–2.75) H: 1.04 (0.59–1.84)	Age, ethnicity, education, family history, smoking, alcohol consumption, sexually transmitted infections, cystitis, prostatitis
Hu et al. (2008), Canada, PCC	1,799/5,039	1994–1997	Range: 20–76	Poultry; 3rd tertile vs. 1 (NA)	0.50 (0.30–0.90)	Age group, province, education, BMI, alcohol use, smoking, vegetable and fruit intake, energy intake
Koutros et al. (2008), USA, Cohort	668/23,080	1993/1997–2003, 8.5y	Range: 40–64	Chicken; 5th quintile vs. 1 (median 42.0 vs. 2.8 g/d)	1.04 (0.78–1.39) H: 1.65 (0.90–3.04)	Age, state of residence, race, family history, smoking status
Li et al. (2008), China, HCC	28/280	1998–2000	Mean (s.d.): case 71.39 ± 6.03, control 71.14 ± 5.78	Poultry; 3rd tertile vs. 1 (≥3 vs. <1 times/w)	1.50 (0.48–4.68)	Education, BMI, smoking, alcohol consumption, food frequency (tomatoes, green vegetables, soybean products, beef, pork and milk); matched by age, place of employment
Richman et al. (2011), USA, Cohort	199/27,607	1994–2008, 14y	Range: 40–75	Poultry; 4th quartile vs. 1 (≥3.5 vs. <1.5 servings/w)	H: 1.15 (0.74–1.78)	Age, energy, BMI, smoking, vigorous activity, lycopene intake, eggs; the following factors were considered and omitted for unsubstantial influence: race, family history, history of diabetes, frequency of PSA screening, use of cholesterol lowering drugs, intakes of (dairy, fish, tomato sauce, fresh tomato products, cruciferous vegetables, calcium, and coffee)
Punnen et al. (2011), USA, HCC	470/512	2001–2004	Case 65.8 ± 8.3, control 65.9 ± 8.5	Poultry; 4th quartile vs. 1 (median 2 vs. 0.25 servings/w)	0.70 (0.49–1.02) H: 0.70 (0.49–1.02)	Age, race, institution, energy intake
Deneo-Pellegrini et al. (2012), Uruguay, HCC	326/652	1996–2004	Range: 40–89	Poultry; 3rd tertile vs. 1 (NA)	0.92 (0.64–1.32)	Age, residence, urban/rural status, education, BMI, family history, total energy intake, other types of meat
Joshi et al. (2012), USA, PCC	1,854/1,094	1997–1997, 1999–2003	Range: 40–79	Poultry; 5th quintile vs. 1 (≥35.7 vs. <7.9 g/1000 kcal/d)	0.80 (0.63–1.01) H: 0.90 (0.60–1.20)	Crude estimate calculated by original data
Mahmood et al. (2012), Pakistan, HCC	195/390	2011	Case 69.77 ± 4.9; control 68.09 ± 5.5	Chicken; 5th quintile vs. 1 (>3 vs. 0 times/w)	0.54 (0.08–3.33)	Ethnicity, socioeconomic status, smoking status, family history, height, physical activity
Stott-Miller, Neuhouser & Stanford (2013), USA, PCC	1,549/1,492	1993–1996, 2002–2005	Range: 35–74	Poultry; 3rd tertile vs. 1 (≥4 vs. <1 times/m)	1.30 (1.04–1.62) H: 1.30 (0.97–1.75)	Age, race, family history, BMI, PSA/DRE tests in previous 5y, education

**Notes.**

Abbreviations are as follows: PCC population-based case-control study HCC hospital-based case-control study H high stage PCa BMI body mass index PSA prostate-specific antigen DER digital rectal examination s.d. standard deviation y years m month w week d day NA not available

a Risk ratio, hazard ratio or odds ratio.

### Main results of meta-analysis

**Figure 2:**
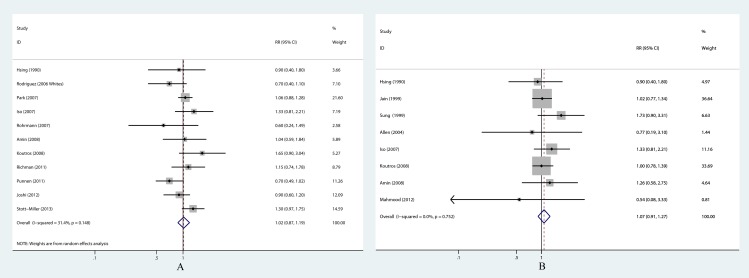
Forest plots of separated groups for poultry consumption and PCa risk. (A) Poultry exposure and high stage PCa risk; (B) Chicken exposure and PCa risk.

**Figure 3:**
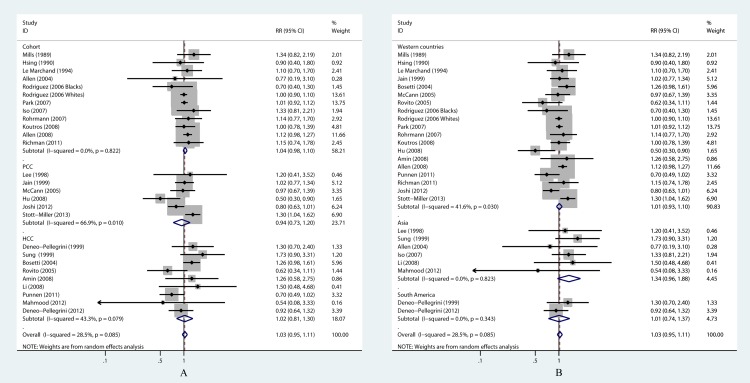
Forest plots of stratified analyses for poultry consumption and PCa risk. (A) Stratified analysis by study design; (B) Stratified analysis by study geography.

In our meta-analysis, no association between poultry consumption and total PCa was observed based on the comparison of extreme quantiles (RR = 1.03, 95% CI [0.95–1.11]) (Table 2 and Fig. 3). The heterogeneity across studies was not statistically significant (*P* = 0.085, *I*^2^ = 28.5%). Pooled RRs from sensitivity analysis did not change substantially, which indicated that the current result was statistically reliable. The shape of funnel plot did not show any evidence of obvious asymmetry. And, no evidence of publication bias was revealed by Egger’s test (*P* = 0.768) and Begg’s test (*P* = 0.868). Separated groups that were restricted to studies with high stage PCa and studies with chicken exposure were synthesized to evaluate the effect on histology-specific PCa incidence and the effect of type-specific poultry. High stage cancer in the present study was comparably defined as Stage IIB or above in the American Joint Committee on Cancer TNM system, although various terminologies (advanced, aggressive or fatal state or grade cancer etc.) may be used in original articles. Pooled result the 11 studies on high stage PCa showed no elevated risk (RR = 1.02, 95% CI [0.87–1.19]) with no significant heterogeneity (*P* = 0.148, *I*^2^ = 31.4%) (Table 2 and Fig. 2A). There were 8 studies identifying chicken as exposure, and null association (RR = 1.07, 95% CI [0.91–1.27]) was acquired in the pooled analysis (Table 2 and Fig. 2B). In sensitivity analysis, the pooled RRs did not change qualitatively after excluding one single study each time. The funnel plot, Egger’s test and Begg’s test proved no publication bias in these separated groups.

**Table 2:** Measures of association between poultry consumption and PCa risk.

Model	Studies	RR (95%CI)	Heterogeneity		Publication bias	
			*P*	*I*^2^ (%)	*P*_Egger's test_	*P*_Begg's test_
Overall	27	1.03 (0.95–1.11)	0.085	28.5	0.768	0.868
High stage PCa only	11	1.02 (0.87–1.19)	0.148	31.4	0.609	0.640
Chicken exposure only	8	1.07 (0.91–1.27)	0.752	0	0.906	0.536
Study design						
Cohort	12	1.04 (0.98–1.10)	0.822	0	0.652	0.837
PCC	6	0.94 (0.73–1.20)	0.010^a^	66.9	0.504	0.707
HCC	9	1.02 (0.81–1.30)	0.079	43.3	0.896	1.000
Geography						
Western countries	19	1.01 (0.93–1.10)	0.030^*^	41.6	0.381	0.484
Asia	6	1.34 (0.96–1.88)	0.823	0	0.138	0.133
South America	2	1.01 (0.74–1.37)	0.343	0	–	–

**Notes.**

* *P* < 0.05;—No available data.

The stratified analysis by study design also showed no elevated risk (Table 2 and Fig. 3A). All of the included studies were divided into three subgroups of cohort studies, case-control studies with population controls and those with hospital controls. The pooled RR of the 12 prospective cohort studies was 1.04 (0.98–1.10), and the heterogeneity among studies was negligible. Similar results were received in the subgroup of hospital-based case-control study. While in the subgroup of population-based case-control study, significant heterogeneity was detected (*P* = 0.010, *I*^2^ = 66.9%). Then a series univariate meta-regression analyses were conducted by adding single covariate at a time, and only the model including adjustment for family history of PCa (yes vs. no) obtained significant result (*P* = 0.128). *τ*^2^ value indicated that this covariate accounted for 71.7% of the heterogeneity. In this subgroup, five of the six studies did not adjusted for family history, and one did (Stott-Miller, Neuhouser & Stanford, 2013). After excluding this study from the analysis, the heterogeneity was no longer significant, and the pooled RR did not substantially change (RR = 0.86, 95% CI [0.69–1.06]; *P* = 0.172, *I*^2^ = 37.3%). Of note, this analysis should be interpreted cautiously for limited studies in the model.

Furthermore, we evaluated the relation between poultry and total PCa in three geographic subgroups (Table 2 and Fig. 3B) according to the region of study population, which were in response to the difference in dietary habits and ethnicities. Countries in North America and Europe, namely the United States, Canada, Italy, etc., were combined in the group of Western countries, because people in these areas had a lot of features in common. Countries and regions in the Asia group comprised of Japan, mainland China, Taiwan and Pakistan. The South America group was merely Uruguay in this meta-analysis. Null associations were obtained in the Asia group and South America group (RR = 1.34, 95% CI [0.96–1.88]; RR = 1.01, 95% CI [0.74–1.37], respectively). Robustness of the result and publication bias in the Asia group were tested, and no statistically significant conclusions were acquired. Pooled result from random-effect model in Western countries also showed that the association was not statistically significant (RR = 1.01, 95% CI [0.93–1.10]), but significant heterogeneity was observed (*P* = 0.03, *I*^2^ = 41.6%). In the univariate meta-regression analyses, none of the pre-specified covariates were related to the strength of the association between poultry and PCa incidence. The Begg’s test, Egger’s test and visual inspection of the funnel plot did not suggest publication bias in Western countries subgroup.

## Discussion

To date, little is known about the etiology of prostate cancer except for a few non-modifiable factors: age, ethnicity/geography and family history. This systematic meta-analysis, based on maximal number of published studies, highlighted the effect of poultry consumption on PCa risk. Studies on total PCa, high stage PCa and chicken exposure were quantitatively synthesized. Stratified subgroups by restricting the analyses to studies with different study designs and in different geographic regions were also performed. Ultimately, we found no evidence of association between them.

Although there were several studies claiming significant effect of poultry on PCa, they couldn’t reverse the holistic conclusion. Punnen et al. (2011) conducted a hospital-based case-control study of 470 cases and 512 controls in the United States. For high stage PCa, they obtained a marginally positive result from a special dataset on grilled and barbecued chicken. But this result was against the result of main dataset on total poultry which was not statistically significant. Another case-control study (Stott-Miller, Neuhouser & Stanford, 2013) also described elevated risk of low stage PCa and total PCa from fried chicken, while the result was not significant for high stage PCa. Thus, the instable results in these articles were self-contradictory and weakened their reliability.

Indeed, the majority of involving studies have found null association regardless of histological stage, exposure type, study design, and study location. Two earlier articles, a dose–response analysis (Norat et al., 2014) and a qualitative review (Cui et al., 2014), were in tune with the present research. The dose–response analysis yielded an RR of 1.01 (95% CI [0.93–1.10]) per 100 g per day increase. And, the qualitative review concluded no association between poultry intake and PCa after comprehensive evaluation. Similarly, a cohort study (Daniel et al., 2011a) also concluded no relationship between them, even the study meticulously considered whether the effect was partly driven by alternative meat using substitution model and addition model. Another two large prospective cohort studies focusing on the relation of poultry consumption to PCa recurrence or progression also failed to get supportive results (Richman et al., 2011; Richman et al., 2010).

We considered several potential reasons for the lack of an overall association between poultry consumption and PCa incidence. First of all, meat mutagens, such as heterocyclic amines (HCAs) and polycyclic aromatic hydrocarbons (PAHs), were the most commonly mentioned mechanism which possibly led to the adverse outcome (Nelson, Demarzo & Yegnasubramanian, 2014). Researchers have voiced their concern about the mutagens in the studies evaluating red meat and processed meat (Mandair et al., 2014). But when it came to poultry, the adverse effect was not significant. For one thing, the carcinogenic effect of the mutagens was dependent on a number of processes, substances and genes. 2-amino-1-methyl-6-phenylimidazopyridine (PhIP), for instance, firstly need to be activated by metabolism, subsequently caused prostate epithelial cell damage and elicited inflammatory response, then formed pro-mutagenic adducts with DNA, and finally triggered mutation and organ-specific cancer (Nakai, Nelson & De Marzo, 2007). Interference to any of the steps by even daily foods and nutrients, such as cruciferous vegetables, would prevent the carcinogenesis (Nelson, Demarzo & Yegnasubramanian, 2014). For another, less personal exposure from poultry compared with red meat also attenuated the risk. Because the carcinogenic by-products primarily generated from high-temperature cooking methods (e.g., grilling, broiling or frying). Also less consumption and lower high-temperature cooking frequency meant less personal exposure (Daniel et al., 2011b). Thus, although the association between PCa and HCAs and/or PAHs generated from white meat have been deeply examined in several studies, little statistically significant results were obtained (Joshi et al., 2012; Koutros et al., 2008; Rohrmann et al., 2015). Another factor that was thought to be responsible for the hazard was heme iron, because it contributed to endogenous formation of carcinogenic N-nitroso compounds. However, poultry was also lower in heme iron than red meat (Cross et al., 2012). Additionally, poultry contained higher amount of unsaturated fat and lower amount of saturated fat compared with red meat (Souci, Fachmann & Kraut, 2008). Epidemiology studies have demonstrated that unsaturated fat tended to lower the cancer risk, while saturated fat increased the risk (Di Sebastiano & Mourtzakis, 2014). Moreover, it was supposed that high poultry eaters often clustered with an overall healthier eating pattern and lifestyle which was characterized by fat-reduced foods, diet foods, and lean meats (Flood et al., 2008). Such an eating habit may further mitigate the risk of PCa.

We revealed evidence of between-study heterogeneity in several subgroups. Whether the study adjusted for family history was proved to be a significant covariate in meta-regression analysis. Prostate-specific antigen (PSA) screening was likely to play similar role. Because, on the one hand, men who enrolled in intensive screening tended to take up healthy lifestyle. On the other hand, screening trial have demonstrated an increase in PCa incidence with PSA test (Hayes & Barry, 2014). However, we could merely extract the information about PSA in a few articles in our reanalysis. Taking the effects of substitution and addition models into account (Daniel et al., 2011a), participants’ energy intake was also under suspicion of confounder. We identified that half of the included studies adjusted for energy intake, but their adjustment methods were various or unavailable. This circumstances hindered us performing further analysis.

Our research had several limitations as with other meta-analyses. Firstly, diverse scales of poultry consumption and definitions of the highest and lowest categories were used in different studies. They acted as obstacles to perform dose–response analysis and caused unambiguous interpretation of the conclusion to some extent. Secondly, food frequency questionnaires were used in all included studies to assess dietary exposure, but some of them did not be validated. In addition, several studies with low quality scores may weaken the test of hypothesis.

## Conclusions

In summary, our comprehensive analysis of available data provides no evidence of association between poultry consumption and PCa incidence. Further well-designed studies are warranted to confirm the result.

## Supplemental Information

10.7717/peerj.1646/supp-1 Table S1 Detailed search strategy for PubMed and Embase

10.7717/peerj.1646/supp-2 Table S2 Quality assessment of included studies based on Newcastle-Ottawa Scale

10.7717/peerj.1646/supp-3 Supplemental Information 3 Supplemental Checklist. Details of the PRISMA 2009 Checklist

## Additional Information and Declarations

### Data Availability

The following information was supplied regarding data availability: All data is available in the manuscript. As a meta-analysis, the data were extracted from published literature and reorganized in Table 1.
