# Supplementary material for: Poultry consumption and prostate cancer risk: a meta-analysis
Source: PeerJ. 2016 Feb 2;4:e1646. doi: 10.7717/peerj.1646 (PMC4741082; doi:10.7717/peerj.1646)
Supplement: Table S1 [file peerj-04-1646-s001.docx]

| Table S1. Detailed search strategy for PubMed and Embase | | |
| --- | --- | --- |
| **Combined PubMed search strategy:** | | |
| #1 | [Poultry](http://www.ncbi.nlm.nih.gov/mesh/68011200)[MeSH Terms] or Poultry[Text Word] or Poultries[Text Word] | |
| #2 | Poultry Products[MeSH Terms] or Poultry Product[Text Word] or Poultry Products[Text Word] | |
| #3 | Domestic Fowl[Text Word] or Domestic Fowls[Text Word] | |
| #4 | Chickens[MeSH Terms] or Chicken[Text Word] or Chickens[Text Word] | |
| #5 | Ducks[MeSH Terms] or Duck[Text Word] or Ducks[Text Word] | |
| #6 | Geese[MeSH Terms] or Goose[Text Word] or Geese[Text Word] | |
| #7 | Turkeys[MeSH Terms] or Turkey[Text Word] or Turkeys[Text Word] or Gallus gallus[Text Word] or Gallus domesticus[Text Word] or Meleagridinae[Text Word] or Meleagrididae[Text Word] | |
| #8 | Meat[MeSH Terms] or Meals[MeSH Terms] or Dairy Products[MeSH Terms] or Dietary Fats[MeSH Terms] or Dietary Proteins[MeSH Terms] or Diet Surveys[MeSH Terms] | |
| #9 | White Meat[Text Word] | |
| #10 | #1 or #2 or #3 or #4 or #5 or #6 or #7 or #8 or #9 | |
| #11 | Prostatic Neoplasms[MeSH Terms] or Prostatic Neoplasms[Title/Abstract] or Prostatic Neoplasm[Title/Abstract] | |
| #12 | Prostatic Cancers[Title/Abstract] or Prostatic Cancer[Title/Abstract] or Prostate Cancers[Title/Abstract] or Prostate Cancer[Title/Abstract] | |
| #13 | Cancer of Prostate[Title/Abstract] or Cancer of the Prostate[Title/Abstract] | |
| #14 | #11 or #12 or #13 | |
| #15 | #10 and #14 | |
| **Combined Embase search strategy:** | | |
| #1 | | poultry*.sh. or poultry*.tw. |
| #2 | | chicken meat.sh. or chicken meat.tw. |
| #3 | | broiler*.sh. or broiler*.tw. |
| #4 | | duck*.sh. or duck*.tw. |
| #5 | | goose.sh. or goose.tw. |
| #6 | | geese.sh. or geese.tw. |
| #7 | | meleagris.sh. or meleagris.tw. |
| #8 | | (Turkey*.sh. or Turkey*.tw.) not Turkey*.pb. not Turkey*.ad. not Turkey*.cp. not Turkey*.cz. |
| #9 | | white meat.sh. or white meat.tw. |
| #10 | | meat.sh. or meat.tw. |
| #11 | | #1 or #2 or #3 or #4 or #5 or #6 or #7 or #8 or #9 or #10 |
| #12 | | prostat* cancer.sh. or prostat* cancer.ti. or prostat* cancer.ab. or prostat* cancer.kw. or prostat* cancer.hw. or prostat* cancer.ot. |
| #13 | | prostat* neoplasm*.sh. or prostat* neoplasm*.ti. or prostat* neoplasm*.ab. or prostat* neoplasm*.kw. or prostat* neoplasm*.hw. or prostat* neoplasm*.ot. |
| #14 | | prostat* tumor.sh. or prostat* tumor.ti. or prostat* tumor.ab. or prostat* tumor.kw. or prostat* tumor.hw. or prostat* tumor.ot. |
| #15 | | prostat* adenoma*.sh. or prostat* adenoma*.ti. or prostat* adenoma*.ab. or prostat* adenoma*.kw. or prostat* adenoma*.hw. or prostat* adenoma*.ot. |
| #16 | | prostat* gland adenoma*.sh. or prostat* gland adenoma*.ti. or prostat* gland adenoma*.ab. or prostat* gland adenoma*.kw. or prostat* gland adenoma*.hw. or prostat* gland adenoma*.ot. |
| #17 | | prostat* duct adenoma*.sh. or prostat* duct adenoma*.ti. or prostat* duct adenoma*.ab. or prostat* duct adenoma*.kw. or prostat* duct adenoma*.hw. or prostat* duct adenoma*.ot. |
| #18 | | prostat* carcinoma.sh. or prostat* carcinoma.ti. or prostat* carcinoma.ab. or prostat* carcinoma.kw. or prostat* carcinoma.hw. or prostat* carcinoma.ot. |
| #19 | | prostat* gland carcinoma.sh. or prostat* gland carcinoma.ti. or prostat* gland carcinoma.ab. or prostat* gland carcinoma.kw. or prostat* gland carcinoma.hw. or prostat* gland carcinoma.ot. |
| #20 | | prostat* intraepithelial neoplas*.sh. or prostat* intraepithelial neoplas*.ti. or prostat* intraepithelial neoplas*.ab. or prostat* intraepithelial neoplas*.kw. or prostat* intraepithelial neoplas*.hw. or prostat* intraepithelial neoplas*.ot. |
| #21 | | prostat* gland tumor.sh. or prostat* gland tumor.ti. or prostat* gland tumor.ab. or prostat* gland tumor.kw. or prostat* gland tumor.hw. or prostat* gland tumor.ot. |
| #22 | | #12 or #13 or #14 or #15 or #16 or #17 or #18 or #119 or #20 or #21 |
| #23 | | #11 and #22 |
