# Supplementary material for: Poultry consumption and prostate cancer risk: a meta-analysis
Source: PeerJ. 2016 Feb 2;4:e1646. doi: 10.7717/peerj.1646 (PMC4741082; doi:10.7717/peerj.1646)
Supplement: Table S2 [file peerj-04-1646-s002.docx]

Table S2. Quality assessment of included studies based on Newcastle-Ottawa Scale

| **Author, Year** | **Design^¶^** | **Selection** | | | | **Comparability** | | **Exposure/Outcome** | | | **Score** |
| --- | --- | --- | --- | --- | --- | --- | --- | --- | --- | --- | --- |
| Mills et al. 1989 | Cohort | ★ | ★ | ★ | ★ | ★ |  | ★ | ★ | ★ | 8 |
| Hsing et al. 1990 | Cohort | ★ | ★ | ★ | ★ | ★ |  | ★ | ★ | ★ | 8 |
| Le Marchand et al. 1994 | Cohort | ★ | ★ | ★ | ★ | ★ |  | ★ | ★ | ★ | 8 |
| Lee et al. 1998 | PCC | ★ | ★ | ★ | ★ | ★ |  |  | ★ | ★ | 7 |
| Deneo-Pellegrini et al. 1999 | HCC | ★ | ★ | ★ |  | ★ | ★ |  | ★ | ★ | 7 |
| Jain et al. 1999 | PCC | ★ | ★ |  | ★ | ★ | ★ |  | ★ | ★ | 7 |
| Sung et al. 1999 | HCC | ★ | ★ |  | ★ | ★ |  |  | ★ | ★ | 6 |
| Allen et al. 2004 | Cohort | ★ | ★ | ★ | ★ | ★ |  | ★ | ★ |  | 7 |
| Bosetti et al. 2004 | HCC | ★ | ★ |  | ★ | ★ | ★ |  | ★ | ★ | 7 |
| McCann et al. 2005 | PCC | ★ | ★ | ★ | ★ | ★ |  |  | ★ |  | 6 |
| Rovito et al. 2005 | HCC | ★ | ★ |  | ★ | ★ |  |  | ★ | ★ | 6 |
| Rodriguez et al. 2006 (Whites & Blacks) | Cohort |  | ★ | ★ | ★ | ★ | ★ | ★ | ★ | ★ | 8 |
| Iso 2007 | Cohort | ★ | ★ | ★ | ★ | ★ |  | ★ | ★ | ★ | 8 |
| Park et al. 2007 | Cohort | ★ | ★ | ★ | ★ | ★ | ★ | ★ | ★ |  | 8 |
| Rohrmann et al. 2007 | Cohort | ★ | ★ | ★ | ★ | ★ |  | ★ | ★ | ★ | 8 |
| Allen et al. 2008 | Cohort | ★ | ★ | ★ | ★ | ★ |  | ★ | ★ | ★ | 8 |
| Amin et al. 2008 | HCC | ★ | ★ |  | ★ | ★ | ★ | ★ | ★ | ★ | 8 |
| Hu et al. 2008 | PCC | ★ | ★ | ★ | ★ | ★ |  |  | ★ | ★ | 7 |
| Koutros et al. 2008 | Cohort |  | ★ | ★ | ★ | ★ | ★ | ★ | ★ | ★ | 8 |
| Li et al. 2008 | HCC | ★ | ★ |  | ★ | ★ | ★ | ★ | ★ | ★ | 8 |
| Richman et al. 2011 | Cohort | ★ | ★ | ★ | ★ | ★ | ★ | ★ | ★ |  | 8 |
| Punnen et al. 2011 | HCC | ★ | ★ |  | ★ | ★ | ★ |  | ★ | ★ | 7 |
| Deneo-Pellegrini et al. 2012 | HCC | ★ | ★ |  | ★ | ★ | ★ |  | ★ | ★ | 7 |
| Joshi et al. 2012 | PCC | ★ | ★ | ★ | ★ | ★ | ★ |  | ★ | ★ | 8 |
| Mahmood et al. 2012 | HCC | ★ |  |  | ★ |  | ★ |  | ★ | ★ | 5 |
| Stott-Miller et al. 2013 | PCC | ★ | ★ | ★ | ★ | ★ | ★ |  | ★ | ★ | 8 |

**^¶^**PCC, population-based case-control study; HCC, hospital-based case-control study
